# Dendrimers in Corneal Drug Delivery: Recent Developments and Translational Opportunities

**DOI:** 10.3390/pharmaceutics15061591

**Published:** 2023-05-25

**Authors:** Anubhav Dhull, Carson Yu, Alex Hunter Wilmoth, Minjie Chen, Anjali Sharma, Samuel Yiu

**Affiliations:** 1 Department of Chemistry, Washington State University, Pullman, WA 99164, USA; anubhav.dhull@wsu.edu (A.D.); alex.wilmoth@wsu.edu (A.H.W.); 2 Center for Nanomedicine, Wilmer Eye Institute, Department of Ophthalmology, Johns Hopkins University School of Medicine, Baltimore, MD 21205, USA; cyu59@jhu.edu (C.Y.); mchen143@jhmi.edu (M.C.); 3 Cornea Division, Wilmer Eye Institute, Johns Hopkins University School of Medicine, Baltimore, MD 21287, USA

**Keywords:** dendrimer, nanotechnology, corneal drug delivery, corneal tissue engineering, targeting, barriers to corneal delivery, corneal diseases

## Abstract

Dendrimers are biocompatible organic nanomaterials with unique physicochemical properties, making them the focus of recent research in drug delivery. The cornea of the human eye presents a challenge for drug transit due to its inherently impenetrable nature, requiring nanocarrier-mediated targeted drug delivery. This review intends to examine recent advancements in the use of dendrimers for corneal drug delivery, including their properties and their potential for treating various ocular diseases. The review will also highlight the benefit of the novel technologies that have been developed and applied in the field, such as corneal targeting, drug release kinetics, treatments for dry eye disease, antibacterial drug delivery, corneal inflammation, and corneal tissue engineering. The review seeks to provide a comprehensive overview of the current state of research in this field, along with the translational developments in the field of dendrimer-based therapeutics and imaging agents and inspire the potential for future developments and translational opportunities in dendrimers based corneal drug delivery.

## 1. Introduction

The development of novel ocular drug delivery systems faces the unique challenge of overcoming the multitude of barrier mechanisms layered throughout the eye’s elaborated physiology [1,2]. The eye houses a defensive architecture that is uniquely rigorous in its impenetrability to foreign matter. Moreover, the layers of highly specialized static and dynamic barriers indiscriminately block the passage of both pathogens and drug molecules into the anterior segment of the eye. In addition to overcoming this array of barriers, corneal drug delivery systems are further tasked with (1) achieving therapeutic drug concentrations localized to target regions in the eye, (2) avoiding side effects in off-target regions, and (3) reducing the burden of treatment on patients [3]. Fulfillment of these criteria continues to pose a major challenge to researchers interested in developing novel ocular drug delivery systems.

Several attempts are currently being made at overcoming corneal drug delivery barriers. Iontophoresis involves using controlled electrical currents as a means of driving ionized drug molecules into tissues [4]. Intracorneal iontophoresis has previously been accomplished in animal models using antibiotics with charged molecular structures, demonstrating its capability to bypass corneal barriers [5,6]. While the treatment achieved therapeutic dosage with limited toxicity in off-target regions, the method relies fundamentally on electric current intensity and duration to enhance drug penetration [6,7]. The avascularity and dense innervation of corneal tissue contribute to the possibility for pain and hypoxia, potentially limiting clinical viability [7]. Sonophoresis, or the use of ultrasound at frequencies 470–880 Hz has shown to increase corneal drug penetration by up to 10-fold when compared to traditional topical delivery, although the precise mechanism of such enhancement remains poorly understood [8]. In vivo animal studies have reported minor structural damage in ultrasound-treated corneal epithelium, likely necessitating further study to confirm safe levels of ultrasound frequency and treatment duration [9]. Microneedles consisting of individual, or arrays of micrometer-sized needles can be applied to inject a wide selection of drugs directly into the corneal stroma [7]. By bypassing the corneal epithelium, microneedle-mediated drug delivery was shown to achieve therapeutic concentrations in the stroma of up to 60-fold higher when compared to topical administration [10]. Although in vivo evaluation of microneedle-mediated administration showed no significant increase in pain, inflammation, or vision loss, one study correlated microneedle treatment to a brief rise in intraocular pressure (IOP) [11]. Despite the promising nature of the new drug delivery methods mentioned earlier, most of them are still in their early stages and have not been extensively utilized for clinical ocular surface drug treatment.

**Figure 1:**
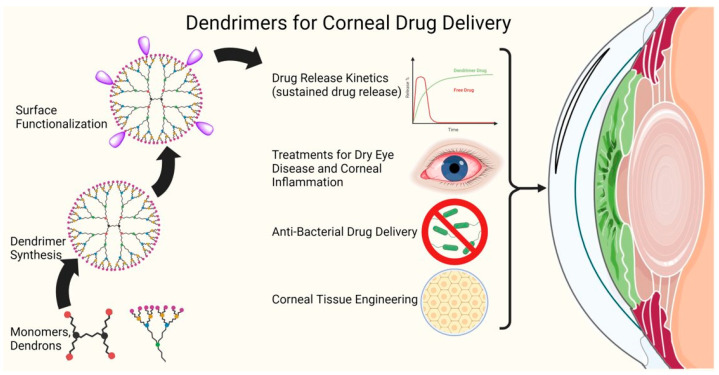
Illustration representing the applications of dendrimers for corneal drug delivery and the treatment of corneal diseases.

Nanotechnology has revolutionized the field of medicine in the past few decades with the development of innovative approaches for targeted drug delivery and diagnosis. One class of nanomaterials that has shown recent promise as a potential ocular drug delivery vehicle are dendrimers, synthetic polymers consisting of repeatable arrays of branch units extending radially from a central core [12]. Branch layers are covalently added in a stepwise fashion denoted by “generations”, allowing for the synthesis of precise molecular architectures that control properties such as size, solubility, and polarity [13,14]. The ability to control for such factors would allow drug molecules to better navigate the eye’s complex physiology. Additionally, modification of branch length and surface functional group identities allows for a highly customizable molecule designed for conjugation to or entrapment of a wide variety of drug molecules [1,13]. The highly customizable nature and physicochemical properties of dendrimers have garnered interest as a candidate for an ocular drug delivery vehicle [1,15]. The past work has been reviewed recently, [16,17] and its application in other tissue has been summarized as well [18]. In this review, the authors focus on dendrimer-mediated drug delivery to the anterior segment of the eye, specifically the most recent developments regarding the treatment of diseases of the cornea (Figure 1).

## 2. Common Diseases of the Cornea

### 2.1. Dry Eye Disease

Dry eye disease (DED) is a common multifactorial disorder of the tear film and underlying ocular surface that currently affects an estimated 46 million Americans [19,20]. Disruptions of the tear film layer cause varying degrees of dryness, which can result in feelings of discomfort, blurry vision, and even functional blindness in severe cases [20,21]. The tear film coats the outer surface of the eye and comprises three distinct layers: the inner mucin layer, middle aqueous layer, and outer lipid layer. DED pathogenesis can be classified by two etiological mechanisms that are closely tied to the functional roles of their respective tear film layers. Evaporative DED is frequently related to dysfunction of the meibomian gland, which normally secretes the lipids of the outer tear film layer that serve to prevent high rates of evaporation of the underlying aqueous layer [19,21]. Aqueous-deficient DED involves deficient production of aqueous tears by the lacrimal gland, which nourish the avascular corneal surface tissue and flush away various debris and foreign bodies [22].

### 2.2. Keratitis

Keratitis is inflammation in the cornea that results from infection by a host of various bacterial, fungal, and protist species. As the structure that forms the eye–environment interface, the cornea faces unique susceptibility to a wide variety of infectious entities. Normally, natural barriers built into the ocular anatomy are sufficient to repulse most pathogens, but compromising these defense mechanisms leaves the eye vulnerable to infection [23]. If such pathogens are allowed into the corneal stroma, inflammation can lead to damage of inner corneal structures responsible for maintaining clear vision [24]. Keratitis can occur as a result of extended contact lens wear or following blunt trauma to the eye [23,25].

### 2.3. Corneal Ulcers

If left untreated, keratitis can progress into a corneal ulcer, or damage to the corneal epithelium associated with infiltration of the underlying stroma [26]. Symptoms include tearing, redness, ocular pain, and potential vision loss if left untreated.

### 2.4. Cogan Syndrome

Cogan’s syndrome is an autoimmune disease characterized by ocular inflammation, frequently in the form of bilateral interstitial keratitis [27]. Most common symptoms include redness, photophobia, tearing, ocular pain, and slow degeneration of visual acuity. Inflammation can sometimes be treated with corticosteroid eyedrops, although its effectiveness is inconsistent [28]. 

### 2.5. Corneal Neovascularization

One of the primary contributors to corneal transparency relates to the avascular nature of the cornea. Upon corneal insult, vascular endothelial growth factor (VEGF) is released by several cell types (epithelial, endothelial, stromal keratocytes), promoting growth of new blood vessels and thus compromising the angiogenic privilege of the cornea [29]. VEGF-A, the primary subclass of VEGF that drives angiogenesis, is the target of numerous drugs. Neovascularization is typically treated using steroids that suppress inflammatory factors in the cornea, including topical formulations such as prednisolone [30]. 

### 2.6. Corneal Fibrosis

Corneal fibrosis is characterized by the formation of vision-obstructing opacities within the stromal layer as a result of injury, infection, or certain surgical procedures involving full or partial removal of the stroma using methods such as photorefractive keratectomy (PRK) [31]. Corneal transparency relies heavily on tight organization of collagen matrices within the stroma allowing light to pass freely through the cornea. Disruption or removal of the ordered collagen structure results in deposition of new, unorganized collagen matrices, thereby obstructing the passage of light and preventing clear vision. 

### 2.7. Conjunctivitis

Conjunctivitis is characterized by inflammation of the transparent membrane (conjunctiva) that lines the inside of the eyelid and the white part of the eyeball, causing redness, itchiness, and in some cases fluid discharge. Conjunctivitis can be divided into infectious and noninfectious categories, prompted by infection or an allergic reaction, respectively. Viruses cause around 80% of all conjunctivitis cases, and although antivirals have shown little effect, artificial tears and cold compresses have shown to reduce some symptoms [32]. Typical treatment of bacterial conjunctivitis involves application of topical antibiotics. While allergic conjunctivitis typically does not adversely affect visual acuity, recurrent symptoms can reduce quality of life for those afflicted [33]. Allergic conjunctivitis can be treated using topical antihistamines.

## 3. Corneal Anatomy and Barriers to Ocular Drug Delivery

### 3.1. Corneal Anatomy

**Figure 2:**
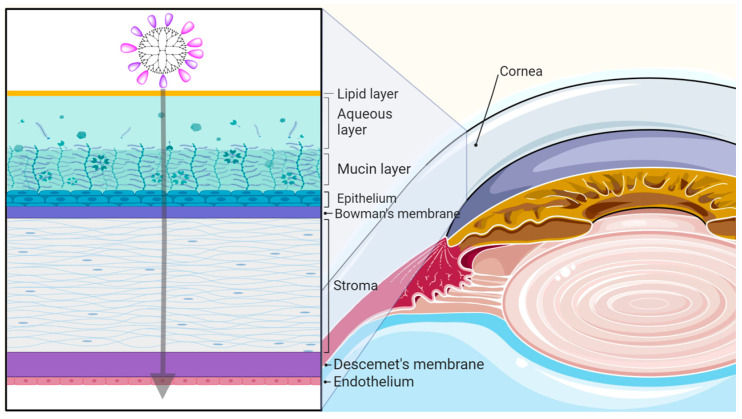
Depiction of the corneal layers along with the passing of dendrimer through the cornea of a human eye.

The cornea is the durable, transparent tissue that, along with the sclera, comprises the superficial anterior segment of the eye. It serves the dual purpose of acting as a barrier to pathogens and debris while also serving as the eye’s strongest refractive component [34]. The precorneal or outer tear film constitutes an outer lipid layer, a middle aqueous layer, and an inner mucin layer (Figure 2). The human cornea is composed of three cellular and two acellular layers, distributed in an alternating fashion. The outermost epithelium is made up of 4–6 layers of nonkeratinized, stratified squamous epithelial cells and serves as the boundary between the eye and the external environment [35]. As such, the outermost layer of cells is regularly shed and replenished to mitigate the effect of damage that can occur from continual exposure to the environment. Between epithelial cells, tight junctions mediated by desmosomes form a watertight seal that prevents tears, and thus pathogens and particulate matter, from entering the eye [36]. Directly posterior to the epithelium is Bowman’s layer, an acellular, nonregenerative meshwork of disorganized collagen fibrils and proteoglycans that constitutes 2–3% of total corneal thickness [34,36,37]. It has been hypothesized that Bowman’s layer plays roles in maintaining corneal structural integrity and in obstructing movement of macromolecules, although neither has been grounded in conclusive evidence [34].

Beneath Bowman’s layer lies the corneal stroma, which comprises 90% of the total corneal thickness and is composed of a network of densely packed collagen fibrils organized into layers known as lamellae. The highly systematic and orthogonal arrangement of lamellae in the stroma allows for the unobstructed passage of light into the eye and is the biggest contributor to corneal transparency [38]. Keratocytes make up the cellular component of the stroma and are sparsely distributed between lamellae [39]. Descemet’s membrane consists of a characteristically banded anterior sublayer and an unbanded posterior sublayer that is continually deposited by the endothelium throughout life. Collectively, both sublayers play a role in maintaining corneal hydration. At the posterior end of the cornea is a single layer of squamous epithelial cells that form the endothelium. The endothelium is equipped with pump mechanisms designed for controlled dehydration of the stroma. Modulating fluid levels in the stroma is vital for maintaining proper transparency and preventing corneal edema [40].

### 3.2. Barriers to Ocular Drug Delivery

**Figure 3:**
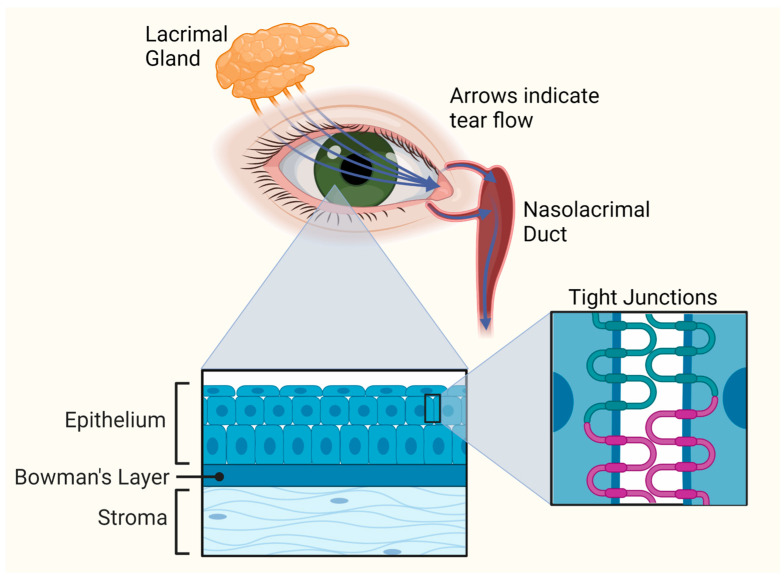
Illustration of the barriers for ocular drug delivery.

There are several types of barriers that ocular drug delivery systems must overcome to achieve therapeutic concentrations in the cornea. These barriers include static (tight junctions between corneal epithelial cells), dynamic (nasolacrimal drainage, blinking), and metabolic barriers (Figure 3). For topically administered drugs, the first barrier is encountered in the precorneal region at the human tear film [41]. The tear film is organized into three layers: an outer lipid layer, a middle aqueous layer, and an inner mucin layer that sits flush against the corneal epithelium and conjunctiva (Figure 2) [42]. The primary function of the outer lipid layer is to reduce tear loss to evaporation, although it passively hinders permeability of hydrophilic drug formulations as well [43]. The middle aqueous and inner mucin layers, sometimes collectively referred to as the mucoaqueous layer, undergo constant drainage and replenishment by the nasolacrimal duct and lacrimal glands, respectively [43]. Such continual replenishment of the tear film drastically reduces bioavailability of topically delivered therapeutic agents. The mucoaqueous layer additionally houses a host of electrolytes, enzymes, and glycoproteins including negatively charged mucins, which can interact with or hydrolyze drug molecules and carriers, further reducing bioavailability [43,44]. The mucoaqueous layer also restricts passage of hydrophobic molecules.

Once past the tear film layer, drug molecules must now bypass the corneal barrier. Similar to the organization of the tear film, the corneal barrier represents a tri-layered barrier that alternates in its affinity to water (hydrophobic–hydrophilic–hydrophobic), further emphasizing the importance of a polarity-controlled formulation to achieve effective penetrance [44,45]. Established primarily by the corneal epithelium, the corneal barrier serves as a mechanical obstruction to pathogens while also limiting fluid loss. Tight junctions (zonula occludens) between epithelial cells selectively regulate paracellular passage of molecules into the cornea, restricting absorption of molecules larger than 10 Å [1]. The lipophilic character of the epithelium establishes an additional barrier to hydrophilic drugs, forming the rate-limiting step of their entry into the eye [45]. The underlying stroma constitutes 90% of the corneal thickness and is hydrophilic in nature, acting as a barrier to lipophilic drug molecules [45]. The endothelium, composed of a single layer of cells, is responsible for maintaining a barrier between the hydrated stroma and the aqueous humor, and poses a lesser barrier to hydrophilic molecules [44].

## 4. Routes of Administration for Corneal Drug Delivery Systems

The avascular nature of the cornea precludes the use of systemic and oral drug delivery routes. Instead, topical administration of therapeutic agents, most commonly in the form of eyedrops, is currently the preferred method of delivery for treating corneal diseases. Topical administration maintains low drug concentrations in the bloodstream while largely mitigating the potential for extraocular side effects. Additionally, topical administration is relatively noninvasive and, with some practice, can typically be applied by patients themselves. Such localization of effect and ease-of-use has heavily contributed to the popularity of topical ocular medications in the current market, with eyedrops making up 90% of all available ophthalmic medications [43].

**Figure 4:**
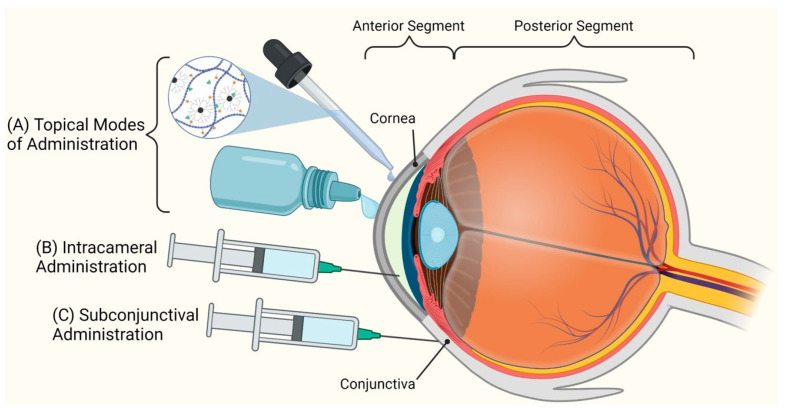
Modes of administration for the drug delivery to anterior segment of the eye: (**A**) topical drug delivery (eyedrops, hydrogels), (**B**) Intracameral injection, and (**C**) subconjunctival injection.

While safe and convenient, use of topical administration suffers most notably from poor bioavailability. Tear drainage through the lacrimal gland during blinking, systemic absorbance of drug through the conjunctiva, and continual tear production (0.5–2.2 μL/min) are all factors that contribute to severely limited drug residence times in the precorneal area [44]. The combined diminishing effects of tear turnover and systemic absorption are responsible for <5% of administered dosages reaching intraocular tissues following eyedrop instillation [45]. Provided such low bioavailability, success of topically administered treatments often requires frequent instillations contingent on strict patient adherence to prescribed regimens. Other routes of administration include subconjunctival and intracameral injections (Figure 4). Subconjunctival injections are considered less invasive when compared to other periocular routes of drug administration [46]. On the other hand, intracameral injection bypasses the ocular barrier and administers drugs directly into the anterior chamber [47].

## 5. Dendrimers for Corneal Drug Delivery

### 5.1. Introduction to Dendrimers 

**Figure 5:**
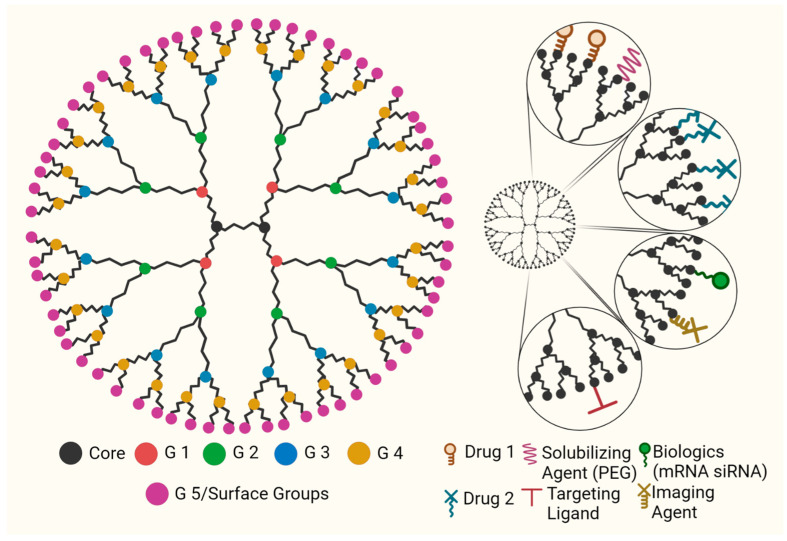
Illustration of the components of the dendrimer (core, surface groups, generations) and the representation of various surface ligands.

Dendrimers are hyperbranched, nanoscale macromolecules whose constituents can be modulated to attain specific, desired chemical properties for a variety of medical applications (Figure 5) [48,49,50]. Vogtle et al. in 1978 [51] synthesized the first hyperbranched molecule, and in the mid-1980s, Newkome [52] and Tomalia [53] synthesized well-defined higher generation dendrimers. The structure of a dendrimer constitutes a multi-armed core, dendron branches, internal voids, and surface groups. The type of the core (number of arms, their alignment) determines the three-dimensional shape and size of the dendrimer whereas the surface groups decide its physiochemical properties. With the addition of each layer, the generation (G) of dendrimer increases with a slight increase in the overall hydrodynamic radius. The number of surface groups increase exponentially with increase in dendrimer generations. Peripheral groups on dendrimers can be further modified to meet the requirements for drug delivery to desired organs, tissues, cells, and subcellular locations. 

There are two classical methods for synthesizing the dendrimers. The first is the divergent approach, used by earlier chemists including Tomalia, Newkome, and Vogtle, in which multiple dendron units with all but one protected site react with a multi-armed core to form the first generation of dendrimer [51,52,53]. The protected sites are then deprotected for further reaction with more dendron units. Purification after each step is required, further increasing the labor and time requirements for this synthesis. Alternatively, the convergent way of dendrimer synthesis, introduced by Fréchet et al. [54], includes multiple branch units combining to form a final dendron that is then attached to the core. Forming a dendron rather than developing inside-out is more advantageous, as it reduces the number of byproducts and purification steps. The stepwise growth of the dendrimer in this synthesis leads to a monodisperse product and allows for designing a macromolecule in accordance with the needs and requirements of the application. To improve yields and efficiency, several approaches such as click chemistry and accelerated divergent techniques have been adopted [55,56]. Dendrimers have been extensively used in biomedical applications [57,58,59,60]. The multivalent surface of dendrimers can be conveniently utilized for the covalent attachment of drugs, biologics, targeting ligands, solubilizing agents, and imaging dyes. The therapeutic agents can be attached to the surface groups with stimuli-responsive linkers to control the drug release [61]. 

### 5.2. Dendrimers for the Treatment of Corneal Diseases

Despite easy access to the surface of eye, drug delivery is a challenge and less than 5% of the dose administered reaches the anterior segment [62]. Poor permeability of the surface tissues and drainage by aqueous and vitreous humor are some of the major challenges to ocular drug delivery [63,64]. In order to increase effectiveness, drugs are applied in high concentrations with repeated dosing causing undesirable ocular and systemic side effects. The corneal drug delivery focuses on the efficiency in the delivery method to increase the residence time of the drug in the cornea to ultimately enhance the bioavailability of the drugs by minimizing the effects of eye discharge. With recent advancements in the field of nanomedicine, various nanomaterials have been used as carriers for ocular and, in particular, corneal drug delivery including polymers, hydrogels, and dendrimers [65,66,67]. The SciFinder search using “dendrimers” and “cornea” as keywords results in over 60,000 references. Key important findings on dendrimer-based formulations for drug and gene delivery to cornea are discussed in this section.

#### 5.2.1. Complexes of Dendrimers with Drugs or Genes for Corneal Delivery

The health of the cornea is highly critical for the maintenance of its transparency and normal vision. Any damage to this may lead to the opacification of the cornea and vision-loss. George et al. evaluated the potential of PAMAM dendrimers for gene delivery for the prevention of corneal allograft rejection. Since tumor necrosis factor (TNF) is reported to be present in significant levels during corneal allograft rejection in animal models, the authors evaluated the dendrimers mediated delivery of soluble TNF receptor immunoglobulin (TNFR-Ig) to corneal endothelium ex vivo using whole thickness rabbit or human corneas. The blockade of TNF activity has been demonstrated to prolong the survival of corneal allografts in animal models [68,69]. The corneas treated with dendrimers and plasmids containing TNFR-Ig genes demonstrated the inhibition of TNF-induced cytotoxicity, suggesting the potential of dendrimer-based gene delivery approach for the prevention of corneal allograft rejection.

Due to constant blinking and rapid drainage of topical eye formulations through the nasolacrimal canal, numerous approaches have been developed to enhance the precorneal residence time and permeability that may eventually reduce the frequency and dose of topical eye formulations. Kompella and coworkers developed G6 dendrimeric polyguanidilyated translocators (DPTs) with triolyl branches and surface guanidine groups as ophthalmic delivery carriers for gatifloxacin (GFX), an antibiotic for the treatment of bacterial conjunctivitis. The recommended effective dose of Zymar eyedrops (0.3% gatifloxacin; Allergan, Irvine, CA, USA) is up to eight times daily, which leads to poor patient compliance. The authors utilized DPTs for sustained delivery of GFX to reduce drug dose and enhance its efficacy. The complexation of GFX with G6DPT enhanced its aqueous solubility and permeability through human corneal epithelial cells, and in turn transport across the isolated bovine sclera–choroid–RPE (SCRPE) layer. DPT-GFX demonstrated two-to-four folds faster killing rate compared to free GFX against anti-methicillin-resistant *Staphylococcus aureus* (MRSA), commonly observed in the conjunctivitis. This enhanced permeability and solubility of DPT-GFX translated to enhanced delivery of GFX to cornea and conjunctiva following a single dose that may allow the decreased frequency of drug administration.

In another report, Molina-Martínez et al. evaluated the potential of water-soluble cationic and anionic carbosilane dendrimers as mucoadhesive polymers for topical delivery of acetazolamide (ACZ), a drug with poor aqueous solubility and limited ocular penetration [70]. An eye drop formulation based on G3 cationic carbosilane dendrimers led to significant improvement in the efficacy in terms of hypotensive effect by reducing the onset time and enhancing the duration after a single instillation. This was attributed to the high-affinity interactions between the cationic carbosilane dendrimers and ocular transmembrane mucins. Keeping the toxicity profile of cationic dendrimer in mind, this is worth mentioning here that the cationic dendrimers were used in very small amount in the formulation and were well-tolerated at the concentrations in the range of 5 to 10 μM. Sun and colleagues utilized PAMAM dendrimers for ocular absorption of Puerarin, an isoflavone compound mostly used in China for the treatment of cataract, glaucoma, and ocular hypertension [71]. The current clinical product, puerarin eyedrops (1%, *w*/*v*) have low bioavailability, low ocular absorption, and short residence time. The use of PAMAM dendrimers PAMAM dendrimers (0.2%) in puerarin formulations showed tolerable toxicity and increased the permeability on excised cornea. In a separate study, Wang and coworkers utilized PAMAM dendrimers to enhance corneal permeation of puerarin using excised rabbit cornea [72].

Lopez et al. [73] further evaluated PAMAM dendrimers using iontophoresis, a noninvasive method for transporting ions through the cornea by application of low electrical gradient, for sustained delivery of dexamethasone to cornea. The study demonstrated that iontophoresis increased the penetration of PAMAM dendrimers deep into the cornea and increased the retention time of the drug in the eye for sustained delivery. It was observed that dexamethasone complexed with G3.5 (anionic) and G4 (cationic) PAMAM dendrimers had enhanced aqueous solubility compared to a free drug and therefore retained longer in the aqueous humor and was delivered more efficiently to the cornea. This increase in efficiency was also attributed to the increased penetration effect of the PAMAM dendrimers, making them an effective drug delivery aid for topical applications in the eye.

#### 5.2.2. Dendrimer Drug Covalent Conjugates for Drug Delivery to Cornea

In addition to physical entrapment or encapsulation of drugs or genes with dendrimers, covalent conjugation of therapeutic agents on the surface of dendrimers using stimuli sensitive linkages for target-specific drug release is a widely used strategy for dendrimer-mediated drug delivery. The covalent conjugation prevents the burst release that occurs with physical encapsulation and provides sustained release of drugs over a prolonged period. 

When stressed due to desiccation, the epithelial cells on the ocular surface show enhanced expression of matrix metalloproteinases (MMPs), proteolytic enzymes that act as markers for DED. For a long time, their inhibitors (MMPIs) promised to be good modulators for abnormal expression of MMPs, but their lack of selectivity in targeting the inflamed tissues has been a major hurdle in their use. To promote site specific drug delivery of MMPIs, Nativi et al. developed a PAMAM based divalent dendron conjugated to two sulfonamidic residues as MMPI inhibitors [74], The resulting divalent inhibitor demonstrated nanomolar binding affinity to MMPs as well as to the transmembrane human carbonic anhydrases (hCAs) XII present in the eye. Furthermore, treatment with divalent inhibitor in a rabbit model of DED resulted in no corneal damage in treated eyes, showed no symptoms of corneal desiccation, and restored normal hydration levels on corneal surface. These results clearly suggest the potential of dendrimer-based MMPIs as potential therapeutics for the treatment of DED.

#### 5.2.3. Dendrimer Polymer Hydrogels for Corneal Drug Delivery and Tissue Engineering

**Figure 6:**
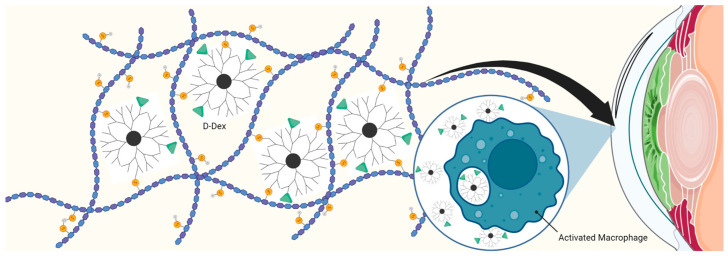
Depiction of Hyaluronic acid crosslinked to hydroxyl terminating G4 PAMAM dendrimers passing through the cornea.

Injectable hydrogels have been widely explored for sustained drug delivery to eye to reduce the frequency of drug administration and increase patient compliance. Kannan et al. investigated the effects of a subconjunctival injectable hydrogel constituting hyaluronic acid crosslinked to hydroxyl-terminating PAMAM G4 PAMAM dendrimers (PAMAM-G4-OH) using photocatalyzed thiolene click chemistry, incorporating dexamethasone (Dex)-conjugated dendrimer (D-Dex) for the treatment of corneal inflammation (Figure 6) [75]. Dex is a highly potent anti-inflammatory agent; however, its repeated instillations are associated with increased intraocular pressure, corneal toxicity, and the development of cataracts [76]. A sustained delivery platform can overcome these issues. Using a mild rat alkali burn model of corneal inflammation, the authors demonstrated that the D-Dex released from the gel after subconjunctival injection targeted activated macrophages in central cornea at diseased location. Moreover, a single subconjunctival injection of the gel containing D-Dex attenuated corneal inflammation and significantly reduced macrophage infiltration in cornea with alkali burn compared to the free Dex, clearly demonstrating the potential of controlled and targeted drug delivery.

Kompella et al. developed a topical formulation of PAMAM dendrimer hydrogel/poly(lactic-co-glycolic acid) (PLGA) nanoparticle platform (HDNP) for the combination delivery of brimonidine and timolol maleate [77]. PLGA nanoparticles are biocompatible and biodegradable and considered safe for formulating therapeutic agents for ocular drug delivery [78]. The HDNP formulation demonstrated slow release of both drugs over a period of 28–35 days in vitro. Moreover, the HDNP formulation could maintain significantly higher concentrations of both drugs in aqueous humor and cornea up to 7 days in comparison to saline, dendrimer and PLGA nanoparticle, clearly demonstrating the capability of enhancing bioavailability and reducing dosing frequency. In another report, Yang et al. developed a mildly crosslinked dendrimer hydrogel (mcDH) by cross-linking G5 PAMAM dendrimers with polyethylene glycol diacrylate (PEG-DA) using an aza-Michael addition reaction for the delivery of brimonidine tartrate [79]. The hydrogel could unionize brimonidine tartrate to encapsulate brimonidine in the form of free base and demonstrated its sustained release and increased corneal penetration. 

Beyond drug delivery, dendrimer hydrogels have also been explored for corneal tissue repair and engineering applications. Corneal wounds are usually repaired with sutures, which are not ideal and may result in infections, corneal inflammation, and neovascularization [80]. Moreover, suturing may result in astigmatism due to uneven wound healing. Dendrimer-based adhesive hydrogels have shown to be advantageous to replace or support sutures for corneal wound healing [81]. Sheardown and colleagues developed G2 polypropyleneimine octaamine dendrimer-based highly crosslinked collagen gels for corneal tissue engineering [82]. The dendrimer-crosslinked gels had superior mechanical properties with Young’s modulus in the range of modulus of natural human cornea. In a separate report by Sheardown et al., the dendrimers in the collagen gel were conjugated to the YIGSR peptide sequence of laminin, still maintaining the overall mechanical properties of the collagen gel [83]. The YIGSR sequence of laminin incorporated into collagen-based scaffold materials has been shown to promote corneal epithelial stratification and regeneration [84]. The results suggested that the YIGSR incorporation using dendrimers into collagen gel promoted the adhesion and proliferation of corneal epithelial cells.

Further advancing the use of dendrimers for corneal tissue applications, Grinstaff et al. used biocompatible dendrimer gels for corneal wound repair [85]. A dendrimer from polyethylene glycol (PEG), glycerol, and succinic acid was synthesized, and the surface was modified using methacrylate groups. The dendrimer ([G1]-PGLSA-MA)_2_-PEG was exposed to visible light to form a crosslinked dendrimer adhesive gel. In an ex vivo experiment using enucleated human eyes with 4.1 mm full-thickness linear incisions in the central cornea, this adhesive gel successfully sealed the wound. Further comparison of results using dendrimers G0–G3, the G1 dendrimer produced the best results. The same group further performed an in vivo study using a chicken model due to its similarity and healing response being similar to a human cornea. The 4 mm full-thickness linear corneal wounds were sealed using photochemical crosslinkable dendritic adhesive hydrogel or 10–0 nylon sutures [85]. Histological studies suggested that the wounds sealed using the adhesive gel healed better than sutures showing a uniform stromal layer with no overlapping of Bowman’s layer.

Most recently, the progress in dendrimer-based corneal tissue engineering has been extended to bioprinting each layer of the cornea [17]. The intention is to develop a biocompatible, low immunogenicity, biodegradable material with suitable crosslinking grid for corneal cell incorporation. Chitosan–collagen polymers have been developed to support corneal epithelium cell growth with good biocompatibility by different groups [86,87]. Tayebi et al. fabricated a biodegradable, transparent scaffold film with chitosan/polycaprolactone polymers, which was compatible with in vitro corneal endothelial cell growth. This chitosan/polycaprolactone 50/25 film was transparent compared to the acellular corneal stroma, which is pivotal to corneal bioengineering. 

In addition to acting as a scaffold for cornea regeneration, dendrimer has the advantage of incorporating pro-regeneration growth factors through sustainable release, especially for physiologically short-lived peptides. Princz MA et al. generated a heparinized epidermal growth factor–dendrimer crosslinked collagen gel. This gel was able to release 90% of the growth factor after 2 weeks and was biocompatible with human cornea epithelium cells [88]. Meanwhile, progress has been made in inhibiting undesired angiogenesis by employing an anti-VEGF dendrimer [89]. A known VEFG blocker aptamer was tethered to form poly(ɛ-lysine) dendrons and were entrapped in a crosslinked methacrylated gellan gum hydrogel. This structure was proven to inhibit in vitro blood vessel invasion and induce its regression. 

Another promising application of dendrimers in ocular disease is to deliver cell-based therapies. Liu Z.J. et al. coated the surface of mesenchymal stem cells (MSC) with an E-selectin PAMAM G5 dendrimer. The infused MSC reached a mouse’s grafted cornea and enhance neovascularization and prompted skin wound healing [90]. Another approach included hybrid nanoparticles embedding a PAMAM dendrimer into an exosome fabricated using sonication to take advantage of the biological properties of exosomes and architectural properties of dendrimers. In total, the G7-NH_2_ showed maximum loading capacity into exosomes, and the size of a loaded exosome was around 150 nm in diameter. The cytotoxicity was reduced to a comparable level of exosome alone and the cellular uptake of the dendrimer was improved significantly. The innovation to “hide” the dendrimer-based drug delivery system and physiological-formed vesicle may provide an advantage in reducing dendrimer toxicity and enhancing drug delivery specificity [91].

#### 5.2.4. Dendrimer-Based Photosensitizers for Photodynamic Therapy

Kataoka et al. synthesized porphyrin dendrimers encapsulated in polyethylene glycol (PEG) shells as photosensitizers for the treatment of corneal neovascularization (CoNV) using photodynamic therapy (PDT) [92]. The current clinical treatment for CoNV involves the use of topical corticosteroids and nonsteroidal anti-inflammatory drugs (NSAIDs) [93]. However, this treatment remains ineffective in the corneas where blood vessels are present for a long time. These blood vessels can be targeted using PDT, which involves the delivery of a photosensitizer to the blood vessels, which can then be excited using a laser to produce reactive oxygen species (ROS) to occlude the targeted blood vessels. Using dendrimer–porphyrin as a photosensitizer encapsulated into polymeric micelles, the authors demonstrated selective accumulation to the pathological vascularized area along with significant regression in neovascularization in PDT-treated mice compared to the dendrimer–porphyrin-free control group. The authors suggested that the selectivity of these photosensitizers to corneal neovasculature could avoid the potential side effects of PDT and might provide a way to deliver drugs selectively to the corneal neovascularized region. Table 1 summarizes the reports on applications of dendrimers in corneal drug delivery and tissue engineering.

## 6. Dendrimer Drug Delivery: Translational Opportunities and Future Outlook

Due to their unique properties as biocompatible nanomaterials, research in dendrimers has seen a recent boom in popularity to solve modern challenges in drug delivery [49,96,97,98,99,100]. The first successful example of a dendrimer-based product is VivaGel^®^, developed by Australia-based company Starpharma. The active ingredient in VivaGel^®^ against sexually transmitted diseases is G4 poly-L-lysine dendrimer, bearing 32 sodium 1-(Carboxymethoxy)naphthalene-3,6-disulfonate groups [101,102,103]. This gel was tested in more than ten clinical trials and the details have been published [104]. VivaGel^®^ is approved in several countries and is available under various brand names. Another poly-L-lysine dendrimer from Starpharma (AZD0466) is undergoing various clinical trials for cancer treatment [105]. USA-based company Ashvattha Therapeutics is developing hydroxyl-terminating polyamidoamine (PAMAM-OH)-based dendrimer therapeutics and imaging agents, several of which are undergoing clinical trials. The first example is OP-101, or D-NAC, which is a covalent conjugate of G4-PAMAM-OH (D) and *N*-acetyl cysteine (NAC). D-NAC, originally preclinically tested for the treatment of neuro- and ocular inflammation, has undergone clinical trials for the safety, tolerability, and treatment of severe COVID-19, with positive interim results (NCT03500627, NCT04321980, NCT04458298) [106,107,108]. Another example is D-4517.2, a conjugate of G4-PAMAM-OH and an analog of sunitinib, an anti-vascular endothelial growth factor (antiVEGF) agent. D-4517.2 has been evaluated in clinical trials for safety, tolerability, pharmacokinetics in wet age-related macular degeneration (AMD) patients or patients with diabetic macular edema (NCT05105607, NCT05387837). The third example from the same company is a dendrimer-based positron emission tomography (PET) imaging agent (18F-OP-801) that is undergoing clinical trials (NCT05395624). These advances in dendrimer therapies symbolize a path towards clinical success. 

With multiple recent ongoing clinical trials on dendrimers and advancements in the chemistry/synthesis of rationally designed dendrimers, the future of dendrimer-based targeted therapies seems very promising. Needless to say, corneal drug delivery is an area that is, in particular, very challenging due to the unique anatomy of the human cornea and can take advantage of the architecture, and physicochemical properties of dendrimers. Their small nanometer size (~2–10 nm) and well-defined globular structure promotes their intracellular uptake across various physiological barriers, including penetration through the corneal epithelium. The multivalent surface not only offers opportunities for drug loading but can also be utilized for imaging applications. Dendrimer-based drugs and imaging agents, due to their high molecular weight, enable improved pharmacokinetics with more specificity to target site, rapid intracellular uptake, higher bioavailability, longer blood circulation time, better in vivo stability, and low off-target toxicity compared to small molecular weight drugs and imaging agents. Moreover, dendrimer nanomedicines offer feasibility of multiple modes of administration for corneal delivery via topical, subconjunctival, and intracameral routes. Less invasiveness and low frequency of dendrimer nanotherapies may further improve patient compliance. For clinical success and commercialization of dendrimer nanomedicines, there is a need to conform to regulatory requirements and chemistry, manufacturing, and control (CMC) standards. Further strong collaborations between dendrimer chemists, formulation scientists, and corneal specialists are highly desired to achieve advancements in optimal drug delivery to/through the cornea.

For successful clinical translation of dendrimer-based therapies, there is a need to develop strategies to overcome any structural limitation they possess. The size of the dendrimers increases with the increase in their generation, so it needs to be thoughtfully chosen depending upon a particular biological application. Dendrimer sizes can vary from 2 to 10 nm and can affect the targeting and clearance [109]. Small-sized dendrimers, generation 5 or lower, can be cleared through the kidneys using glomerular filtration; however, higher generation dendrimers may accumulate in the liver for hepatic clearance [60,110]. The properties of dendrimers are governed by their surface groups, which can be altered by changing the surface chemistry. For example, the cationic dendrimers are toxic and cause cell lysis due to their interaction with cell membranes [111]. Kannan et al. had great success in utilizing neutral, hydroxyl-terminating PAMAM dendrimers for targeted treatment of inflammation in severe COVID-19 and brain and ocular diseases [60,100,107,112]. Moreover, the controlled synthesis of dendrimers allows their rational design to create clinically relevant dendrimers for various drug delivery applications [96,113]. 

The nature of the cornea in the human eye to act as an inherently impenetrable barrier creates the challenge for drug transit into the interior segments of the human eye. In this review, we focused attention on the anatomy and physiology of the cornea causing these drug delivery challenges and some of the diseases associated with the cornea of the human eye. The unique properties allowing dendrimers to bypass the natural barrier, as well as the novel technologies in which recent investigations have applied the dendrimers, such as in (1) corneal targeting, (2) drug release kinetics, (3) treating dry eye disease, (4) anti-bacterial drug delivery, (5) treating corneal inflammation, and for (6) corneal tissue engineering have been discussed. The recent success in the field of dendrimers with several dendrimer-based therapeutics or imaging agents being tested in human clinical trials clearly depicts the emerging opportunities in this field for targeted drug delivery applications to address unmet medical needs. As time moves on, new discoveries using dendrimer technologies will improve the efficacy and efficiency of current medical treatments as well as allow for new solutions to ocular diseases.

## Figures and Tables

**Table 1:** Summary of the studies using dendrimers for corneal drug delivery and tissue engineering.

S. No.	Study	References	Type of Dendrimers Used
1.	Increased penetration of drugs through cornea using dendrimers	Brobeck et al. [94]	PAMAM dendrimers of various generations.
		Lopez et al. [73]	Generation 3.5 and generation 4 PAMAM dendrimers.
		Kompella et al. [77]	PLGA-PAMAM hydrogel dendrimer Complex.
		Yang et al. [79]	Generation 5 PAMAM dendrimer.
2.	Treatment of dry eye disease (DED)	Nativi etal. [74]	Generation 2 PAMAM dendrimer.
3.	Anti-bacterial drug delivery to cornea	Kompella et al. [95]	Generation 6 dendrimeric polyguanidilyated translocators (DPTs)
4.	Treatment of corneal inflammation	Sun et al. [71]	Generation 3 PAMAM dendrimers with Puerarin
		Kannan et al. [75]	Generation 4-hydroxyl terminating PAMAM dendrimer.
5.	Corneal tissue engineering	Sheardown et al. [82]	Generation 2 polypropylene octamine dendrimers
		Grinstaff et al. [85]	(PGLSA-MA)_2_-PEG dendrimer
		Sheardown et al. [83]	Generation 2 polypropyleneimine dendrimer.

## Data Availability

No new data is created. Data sharing is not applicable.
